# Out of Sight: Sex Differences in Public and Semi-Public Drug Use Settings Among People Who Use Opioids in Baltimore, Maryland

**DOI:** 10.3390/ijerph23040534

**Published:** 2026-04-20

**Authors:** Carl A. Latkin, Lauren Dayton, Ananya Bhaktaram, Melissa A. Davey-Rothwell, Haley Bonneau, Grace Tian Yi, Oluwaseun Falade-Nwulia

**Affiliations:** 1 Department of Health, Behavior and Society, Bloomberg School of Public Health, Johns Hopkins University, Baltimore, MD 21205, USA; 2 Division of Infectious Diseases, School of Medicine, Johns Hopkins University, Baltimore, MD 21218, USA

**Keywords:** overdose, setting, gender, sex differences, harm reduction, HIV, stigma, drug use, opioids, homelessness

## Abstract

**Highlights:**

**Public health relevance—How does this work relate to a public health issue?**

- Drug use settings are where overdose, infectious disease transmission (HIV/HCV), and other drug-related harms occur.
- Identifying the specific settings and the attributes of individuals who use opioids in these settings can help the development of settings-based interventions.

**Public health significance—Why is this work of significance to public health?**

- Quantifies sex differences in drug use locations across nine setting types in a large community sample.
- Provides epidemiological information on setting use and examines social and structural factors that shape drug use setting choice and advances a settings-focused framework for equitable overdose prevention.

**Public health implications—What are the key implications or messages for practitioners, policy makers and/or researchers in public health?**

- Prioritize harm reduction interventions that target drug use settings with trainings and materials.
- Include gender-responsive designs in harm reduction and evaluate setting-specific strategies to reach women who may avoid public drug use.

**Abstract:**

Background: Drug use settings are critical determinants of overdose risk and other drug-related harms. Although sex differences in drug use patterns are well documented, less is known about sex differences in the types of locations where people use drugs. This study examined sex differences in drug use settings among people who use opioids. Methods: Data were from the baseline survey of the OASIS project, a community-based study conducted in Baltimore, Maryland (N = 869), focusing on 9 specific types of locations where participants reported drug use in the past 30 days: their own residence, someone else’s residence, street, alley, park, abandoned building, public restroom, car, and other locations. Bivariate and multivariable logistic regression models examined associations between sex and drug use settings, adjusting for age, race, education, homelessness, and frequency of drug use. Results: The sample included 346 women and 523 men. In adjusted models, women had significantly lower odds than men of using drugs on the street (aOR = 0.49, 95% CI 0.35–0.70), in alleys (aOR = 0.50, 95% CI 0.35–0.69), parks (aOR = 0.57, 95% CI 0.42–0.78), abandoned buildings (aOR = 0.53, 95% CI 0.38–0.75), cars (aOR = 0.55, 95% CI 0.41–0.73), and other locations (aOR = 0.59, 95% CI 0.37–0.94). Sex was not significantly associated with drug use at one’s own residence or someone else’s residence. Conclusions: Women who use opioids were significantly less likely than men to use drugs in public and semi-public settings, which may reflect gendered patterns of stigma, interpersonal violence, and safety concerns. Harm reduction programs should focus on making current drug use settings safer and developing additional safer settings with an emphasis on addressing barriers for women to access harm reduction services, including women-centered overdose prevention centers and household-based overdose response training.

## 1. Introduction

In many countries, opioid overdose deaths have increased dramatically. In Canada, from 2018 to 2023, opioid overdose fatalities increased by approximately 155% (4.7 to 12.0 per 100,000) [1]. Additionally, both Australia and Scotland have also seen increases in opioid fatalities [2]. In 2024, Scotland reported 27.1 drug misuse deaths per 100,000 for males and 11.6 deaths for females, with 80% involving opioids [3]. Although men account for a larger absolute number of overdose deaths, overdose mortality rates among women in the U.S. have risen sharply over the past two decades. In the U.S., from 2003 to 2023, the age-adjusted opioid overdose death rate increased from 2.8 to 13.6 per 100,000 among females, with over 20,000 opioid-related deaths among women in 2023 [4].

Social ecological and multilevel models of substance use-related health behaviors have emphasized the importance of settings where drug use occurs. Settings where illicit opioids and other drugs are used play a critical role in shaping drug-related harms, especially fatal overdoses and transmission of blood-borne infectious diseases. Two popular theoretical approaches for understanding the role of drug use settings are the risk environment framework [5] and the drug, set, and setting framework [6]. Settings shape the embedded interactions among social, psychological, and physiological factors that influence substance use behaviors.

Previous studies have found that settings where drugs are used are associated with risk behaviors, infectious disease transmission, and overdose mortality [7,8,9,10,11,12,13]. Injection drug use in specific types of settings, such as shooting galleries, has been associated with HIV and HCV risk behaviors and HIV serostatus [13,14]. Although non-residential drug use settings have been linked to higher infectious disease transmission, research suggests that many fatal overdoses occur in private housing environments and that naloxone is less likely to be administered in these settings compared to more public settings [15]. Location of drug use has been documented to be associated with fatal overdose risk. Solitary drug use increases the risk of fatal overdose, as it minimizes the opportunity for emergency service response or bystander intervention [16,17]. Analyses of overdose death investigations indicate that many overdose decedents are found without an immediately available bystander, frequently in housing/private residences [15]. To develop drug setting-focused interventions, it is essential to understand who frequents and avoids specific types of drug use settings and the prevalence of drug use in different settings.

Sex differences in drug use patterns and barriers to drug treatment are well documented [18]. However, there is little extant research on how sex may influence the specific types of drug use settings and compound the risk of drug use in these locations. Several factors suggest that there may be gender-based differences in the settings where people use opioids. Qualitative studies suggest that women may be more likely to avoid using in public settings [16,19]. Perceived safety, fear, and stigma concerns about using in unpredictable social settings have been found to be key drivers for women using alone [20]. Women are also more likely than men to use drugs with an intimate partner rather than in larger peer networks [21,22]. If women co-reside with a partner, then the patterns of drug use may shift toward private residential settings rather than public drug use.

Women who use drugs face both symbolic and structural violence through multiplicative forms of stigma from gendered expectations of being a woman, substance use, and frequent co-occurrence with poverty, sex work, criminalization, and HIV/HCV status [23,24,25]. A systematic review of 75 studies of drug use stigmatization found that qualitative studies consistently demonstrated that women are more likely than men to be stigmatized for drug use [24]. The qualitative findings highlight that women who use drugs experience compounding stigmatization, in part, due to gendered expectations of womanhood, motherhood, and moral failures centered around ideas of the associations of promiscuity, cleanliness, and substance use [24].

Bourdieu’s concept of symbolic violence describes the process through which social and cultural practices are reinforced within a person’s milieu or social environment [26]. Women report intense stigma related to drug use, particularly if they are visibly pregnant or are known to be mothers [22,27,28]. Many women report internalizing drug use stigma as a form of gendered failure, making them more likely to self-isolate from support networks to conceal drug use [25,27]. Due to these multiple and reinforcing sources of social stigma, women who use drugs may choose to use indoors or in mostly private settings, as using indoors reduces visibility and the risk of social sanction or Child Protective Services (CPS) involvement.

Moreover, a systematic review also found that women who use opioids report higher rates of both lifetime and past year interpersonal violence (IPV) than women who do not use opioids and men [22]. Previous qualitative studies have also shown that women who use drugs may face gender-based violence, including sexual assault, robbery, and intimate partner violence, when using in public settings [29,30]. Consequently, concern about violence may differentially impact the choice of drug use settings among men and women.

Given the public health importance of drug use settings, supervised injection sites and overdose prevention centers (OPCs), which have personnel available who can quickly respond to and reverse and overdose, have been implemented to reduce overdose, HIV, HCV, and bacterial infection risks. There is substantial data on the public health benefits of these settings. The first publicly sanctioned OPC in the U.S. opened in New York City in 2021 and has reversed thousands of overdoses with no on-site deaths [31]. However, women accounted for only 20% of clients. Studies in Canada suggest that while OPCs were seen as “safe havens,” they are often viewed as masculine spaces as men frequently dominate them, and women who accessed them were routinely subjected to harassment. Some expressed fear that the harassment would escalate to physical violence or prevent them from future access [29]. Given the barriers for women to utilize OPCs, it is important to gain a greater understanding of the settings where women do use drugs to enhance gender-informed harm reduction programs.

In addition to gender, other structural factors may influence where people use opioids and other drugs. Among those who inject drugs, experiencing homelessness has been found to be associated with injecting in public [11]. For people experiencing homelessness, not only do they not have their own place to use drugs, but they may not have the same access to other people’s private residences for drug use, and thus are forced to use in public settings. Individuals who use drugs more frequently may also be more likely to use in a greater number of settings. Moreover, the frequency of drug use is also an indicator of the severity of substance use disorder. Heightened substance use disorder often corresponds with greater withdrawal symptoms. To relieve withdrawal symptoms, individuals may use drugs immediately after purchasing them, leading them to use in public or non-residential settings.

Identifying factors associated with using drugs in specific types of settings can help in the development of setting-based approaches to mitigate the harms associated with opioid use. In the current study, we examined correlates of setting-specific drug use, with a primary focus on sex differences among people who use opioids, who reported that they had used drugs in the prior month. Much of the prior research on settings has been qualitative and has lacked a detailed analysis of who uses drugs in specific settings. Such information can help guide gender-informed harm reduction interventions for people who use opioids.

## 2. Methods

### 2.1. Study Design and Participants

Data for this analysis were drawn from the baseline survey of the OASIS (Overdose and Settings Investigation Study) project, a community-based study conducted in Baltimore, Maryland. The OASIS study was designed to identify spatial patterns of drug overdose events and to train people who use drugs as peer educators for distributing naloxone and HIV/hepatitis C prevention supplies in locations where drug use occurs. Recruitment was conducted through street outreach in neighborhoods with elevated rates of illicit drug activity. We also recruited from community services such as soup kitchens and prioritized recruiting a substantial proportion of women into the study. To be eligible, individuals had to (1) be at least 18 years old, (2) have used illicit opioids within the preceding 30 days, and (3) reside within the greater Baltimore metropolitan region. The Institutional Review Board at the Bloomberg School of Public Health approved all procedures. Each participant was compensated $40 upon completion of the study visit. From April 2023 through July 2025, 873 individuals enrolled and completed the face-to-face baseline assessment.

### 2.2. Measures

#### 2.2.1. Drug Use Settings (Outcomes)

Participants indicated whether they had used drugs in each of nine specific locations during the past 30 days. The survey item stem read: “In the past 30 days, did you use drugs at:” followed by response options for “(1) your house, (2) someone else’s house, (3) street, (4) alley, (5) park, (6) abandoned building, (7) public restroom, (8) car, and (9) other.” Participants who reported using in “other” settings were asked to describe those settings. Each setting was then analyzed as a separate binary outcome (yes/no). After participants responded to the 9 drug use location questions, they were asked to report the location where they used most often in the past month via the question “Please think about the location where you used drugs most often in the past month. What type of location is this?”

#### 2.2.2. Sociodemographic Characteristics

Participants provided self-reported information on sex assigned at birth (male or female). Additional sociodemographic characteristics included: age (continuous), sexual orientation, race (African American/Black, White, Asian/Other/Refused), and educational attainment. Education was collapsed into two categories: high school/GED completion or less versus any post-secondary education. Recent housing instability was assessed by asking whether the participant had spent at least one week in the past six months sleeping in locations such as on the street, in a park, in an abandoned structure, in a vehicle, in an emergency shelter, or while squatting. Those who endorsed any of these experiences were classified as having experienced recent homelessness.

#### 2.2.3. Substance Use Frequency

The survey assessed lifetime and recent use of heroin, powder cocaine, crack cocaine, methamphetamine, buprenorphine, and fentanyl. Participants who reported any use of a given substance in the prior 3 months were asked how often they used it, with response options including never, less than once per month, monthly, weekly, or daily/almost daily. These frequency categories were adapted from the 2019 European School Survey Project on Alcohol and Other Drugs (ESPAD) instrument [32]. Crack and powder cocaine were combined into a single cocaine variable; participants reporting daily or near-daily use of either form were classified as high-frequency cocaine users. A parallel dichotomous variable was created for heroin/fentanyl, with daily or near-daily use of either substance defining high-frequency opioid use. Recent injection drug use was assessed with the question “When was the last time that you injected drugs?” The nine response categories ranged from “never” to “within the past day”. A dichotomous variable was then created: “within the past month” and “not within the past month.”

#### 2.2.4. Statistical Analysis

The nine drug use setting variables served as the primary outcomes. We first computed descriptive statistics and chi-square tests and then estimated bivariate logistic regression models to examine unadjusted associations between each predictor and each setting outcome. Predictors with *p* < 0.15 in bivariate analyses were included in the multivariable logistic regression models [33]. We report odds ratios (ORs) and adjusted odds ratios (aORs) with corresponding 95% confidence intervals (CIs). Four participants with incomplete data were excluded, yielding a final analytic sample of 869. Analyses were performed using Stata version 17 [34].

## 3. Results

The sample included 346 women and 523 men. There were few demographic differences based on sex (Table 1). Overall, the two groups were similar in age (48.6 years, women vs. 49.6 years, men) and educational attainment, with 72.0% of women vs. 75.1% of men having less than or equal to a high school degree (12th grade) or a GED. Homelessness in the past six months was also comparable (41.0% of women vs. 46.3% of men). There were significant sex differences in race/ethnicity. Women were somewhat more likely than men to identify as White (28.9% vs. 22.4%), whereas men were more likely to identify as African American/Black (72.7% vs. 64.2%).

Most participants reported using drugs in residential locations, either their own house (80.7%), someone else’s house (70.1%), or both locations (54.7%). Only 3.9% reported neither location. The mean number of drug use locations reported was 4.8 (SD = 2.3). The majority of participants (85.8%) reported using drugs in the prior month in a non-residential location. In addition to reporting on their use in the 9 drug use settings in the prior 30 days, participants also reported on the most frequently used setting. Although own house (47.3%) and someone else’s house (14.2%) were reported as the most frequently used locations, 38.5% reported other settings as their most frequently used locations, with street (11.1%), alley (9.2%), and abandoned building (10.1%) having similar proportions.

For use in non-residential settings, the most frequently reported settings were street (74.3%), alley (60.5%), park (51.0%), car (49.6%), abandoned building (47.3%), public bathroom (37.4%), and other settings (11.5%). Participants who reported another drug use setting were asked to describe the settings, and over 30 settings were listed. Three or more participants reported drug use in the following settings: backyard, subway/light rail, garage, bus/bus stop, parking lot and parking garage, woods, hospital, church, and backyard. Only one participant explicitly mentioned a shooting gallery.

Sex differences were pronounced for locations of drug use. Men were significantly more likely than women to report using drugs on streets (78.6% vs. 67.9%, *p* < 0.001), in alleys (64.2% vs. 54.9%, *p* < 0.01), parks (55.3% vs. 44.5%, *p* < 0.01), abandoned buildings (50.9% vs. 41.9%, *p* < 0.01), cars (55.4% vs. 40.8%, *p* < 0.001), and “other” locations (13.4% vs. 8.7%, *p* < 0.05). Use of public restrooms was marginally significant, with higher rates among men (39.8% vs. 33.8%, *p* < 0.10). Use at one’s own house (83.5% of women vs. 78.8% of men) and use at someone else’s house (68.2% of women vs. 71.3% of men) did not differ significantly by sex.

### Logistic Regression Models

In bivariate logistic regression models (Table 2a), gender was consistently associated with drug use setting. Compared with males, females had lower odds of reporting drug use in most public/outdoor locations, including streets, alleys, parks, abandoned buildings, cars, and “other” settings. Associations for drug use in public bathrooms suggested a similar pattern. In contrast, there were no statistically significant gender differences in reported use at one’s own house or at someone else’s house in the unadjusted models.

Several other participant characteristics were associated with reported drug use setting in the bivariate logistic regression models. Homelessness in the past 6 months showed the strongest and most consistent associations: homelessness was associated with substantially lower odds of using at one’s own house and substantially higher odds of using in someone else’s house and in multiple public/outdoor locations (street, alley, park, abandoned building, public bathroom, car, and “other” settings). Increasing age was associated with higher odds of using at one’s own house and lower odds of using in public/outdoor settings, particularly streets, alleys, parks, abandoned buildings, public bathrooms, cars, and “other” settings.

In unadjusted models, race was also associated with setting. Relative to Black participants, White participants had higher odds of using in several public/outdoor settings (street, alley, park, abandoned building, and public bathroom), while associations for using in one’s own house or someone else’s house were weaker. Participants in the “other” race category also demonstrated elevated odds for some public settings (e.g., alleys, public bathrooms, and cars). However, estimates were generally smaller and less consistent than those for White participants.

Daily drug use patterns were related to setting in bivariate analyses. Daily heroin/fentanyl use was associated with higher odds of using in multiple public/outdoor settings (e.g., someone else’s house, street, alley, park, abandoned building, public bathroom, and car), whereas daily crack/cocaine use showed particularly strong associations with using in public settings such as streets, alleys, parks, and abandoned buildings. A higher level of education (>12th grade/GED) was not associated with most settings in bivariate models, except for higher odds of reporting use in “other” settings.

In adjusted models (Table 2b), gender differences remained pronounced and largely unchanged. Females had significantly lower odds than males of reporting drug use in public/outdoor settings, including streets (aOR = 0.49, 95% CI: 0.35–0.70), alleys (aOR = 0.50, 95% CI: 0.35–0.69), parks (aOR = 0.57, 95% CI: 0.42–0.78), abandoned buildings (aOR = 0.53, 95% CI: 0.38–0.75), cars (aOR = 0.55, 95% CI: 0.41–0.73), and other locations (aOR = 0.59, 95% CI: 0.37–0.94). The association between gender and using at one’s own residence did not reach statistical significance (aOR = 1.34, 95% CI: 0.93–1.92). Gender and drug use in a public bathroom was marginally associated (aOR = 0.76, 95% CI: 0.56–1.03), and gender was not retained in the multivariable model of using at someone else’s residence.

In multivariable logistic regression models, homelessness was a consistent predictor of drug use setting. Compared with participants not experiencing homelessness in the past 6 months, those reporting homelessness had substantially lower odds of using at their own residence (aOR = 0.42, 95% CI: 0.29–0.60), and markedly higher odds of using at someone else’s residence (aOR = 2.37, 95% CI: 1.69–3.34) and in public/outdoor locations, including streets (aOR = 3.82, 95% CI: 2.54–5.74), alleys (aOR = 3.78, 95% CI: 2.68–5.34), parks (aOR = 3.33, 95% CI: 2.44–4.55), abandoned buildings (aOR = 6.11, 95% CI: 4.39–8.50), public bathrooms (aOR = 2.44, 95% CI: 1.78–3.33), and cars (aOR = 1.57, 95% CI: 1.16–2.12).

Age was also associated with setting. Older age was associated with lower odds of using on streets (aOR = 0.96, 95% CI: 0.95–0.98) and in alleys (aOR = 0.97, 95% CI: 0.95–0.99), with similar inverse associations observed for abandoned buildings, public bathrooms, and cars (aORs ~0.98–0.99). Race was associated with several public settings. Compared with Black participants, White participants had higher odds of reporting drug use on streets (aOR = 1.80, 95% CI: 1.06–3.06), in alleys (aOR = 2.97, 95% CI: 1.84–4.80), abandoned buildings (aOR = 2.43, 95% CI: 1.58–3.74), and public bathrooms (aOR = 1.70, 95% CI: 1.17–2.48), and marginally associated with drug use in parks (aOR = 1.41, 95% CI: 0.95–2.09). Participants of “other” races did not show statistically significant differences across settings (vs. Black), although the estimate for public bathrooms suggested a possible trend (aOR = 1.79, 95% CI: 0.97–3.30).

Substance use frequency was associated with settings. Daily heroin/fentanyl use was associated with increased odds of using in alleys (aOR = 1.51, 95% CI: 1.04–2.20), parks (aOR = 1.47, 95% CI: 1.03–2.10), and public bathrooms (aOR = 1.69, 95% CI: 1.16–2.47); the association with car use was marginal (aOR = 1.39, 95% CI: 0.99–1.96). Daily crack/cocaine use was associated with increased odds of using at someone else’s residence (aOR = 1.72, 95% CI: 1.25–2.36), in an alley (aOR = 2.25, 95% CI: 1.61–3.13), in a park (aOR = 1.72, 95% CI: 1.27-2.34), and in an abandoned building (aOR = 2.30, 95% CI: 1.65-3.21); estimates for other settings were smaller and not statistically significant (e.g., public bathroom: aOR = 0.97, 95% CI: 0.71–1.33; car: aOR = 1.14, 95% CI: 0.85–1.52; other: aOR = 1.39, 95% CI: 0.88–2.18). Finally, education >12th grade/GED (vs. ≤12th Grade/GED) was associated with higher odds of reporting drug use in “other” settings (aOR = 1.71, 95% CI: 1.09–2.68).

A post hoc analysis suggested that for those who experienced homelessness in the prior six months, there was a much greater sex difference in using on the street and parks compared to those who were housed. In contrast, there was a greater sex difference in drug use in a car for those who were housed compared to those who had experienced homelessness.

## 4. Discussion

Even after adjusting for key drug use and demographic variables, we found that women were significantly less likely than men to use opioids in six of the seven settings outside of their residence or someone else’s residence. Moreover, the seventh setting of drug use in public bathrooms was marginally associated with female sex. These findings fit with prior qualitative research suggesting that women who use drugs are more likely to avoid public drug use, which may be due in part to IPV and stigmatization [16,19].

Homelessness in the prior six months was the most consistent and strongly associated factor with public drug use and was negatively associated with drug use at one’s own home. Close to half (44%) of the sample reported homelessness in the prior six months. To use drugs in a residential location other than one’s own, there’s often a need to have network members who will share their residence for drug use. Unhoused individuals may have no or limited access to such networks or reside in areas far from their network members. Given the much greater odds of public drug use among those who experienced homelessness, programs and facilities such as overdose prevention centers are especially needed for this population. Future research should examine in more detail how individuals experiencing homelessness may be able to use drugs in safer settings.

Although there was no statistical difference in the proportion of women and men who reported experiencing homelessness in the prior six months, women who use drugs and experience homelessness tend to be more vulnerable to violence and maltreatment. Unhoused women, compared to housed women, may be more stigmatized, as it may be more difficult for them to conceal their drug use. Prior research suggests that women who use drugs are more dependent on partners than men [21]. Homeless women may be even more dependent on partners, which may make it difficult to modify their drug use patterns. The dynamic of women drug users’ dependency on male partners can be especially problematic for drug treatment since treatment programs rarely work with couples. In addition to providing medications for opioid use disorder (MOUD), there is a substantial need to develop and evaluate treatment programs that offer couples counseling within a harm-reduction framework.

Women who use opioids may have multiple marginalized identities that may interact through social structures and power dynamics to symbolic, interpersonal, and structural forms of violence, resulting in syndemic vulnerability leading to multiple poor health conditions. Examining the experiences of women who use opioids through the theoretical lenses of intersectionality and Bourdieu’s theory of social reproduction and symbolic power allows for greater understanding of the social and local contexts that guide behavior and risk assessment. Women who use opioids who are unhoused may experience the stigma of their multiple identities of being a drug user, female, and unhoused. Housing is a key resource associated with poor health care engagement, and the lack of it can perpetuate stress and distress through lack of access to other resources and dramatically increase vulnerability to physical and psychological harms.

Four of the non-residential settings were also associated with drug use frequency, and injection drug use was associated with three of the non-residential settings. Though more frequent drug use would increase the probability of drug use in more settings, it is also an indicator of the severity of opioid use disorder and is likely to be associated with the severity of withdrawal symptoms. One potential driver of public drug use is the need to use drugs immediately after purchase to alleviate withdrawal symptoms. Future research should focus on developing effective methods to reduce and cope with withdrawal symptoms, which may reduce the urgency to use opioids, especially immediately after purchase.

Most participants reported using drugs in several settings. Given these findings, overdose prevention programs should delineate and target drug use settings and their communities. Most of the respondents who used at residences also used in non-residential settings. Future research should examine how interventions can be adapted to a range of drug use settings. However, drug use setting types may differ by and within locale. For example, in Baltimore, Maryland, there is a large stock of abandoned buildings where people use drugs. These abandoned buildings may differ in their level of privacy and access to harm reduction supplies. If the buildings are used frequently, there is more likely to be individuals on hand who can respond to an overdose. However, more people frequenting a setting reduces privacy and control over the space. Moreover, when others are present, there may be an increased sense of urgency to quickly use drugs, which can contribute to overdose. Future research and harm reduction programs should examine how people who use drugs can configure spaces and socially organize these spaces to prevent victimization and increase the availability of naloxone and other harm reduction materials, as well as develop plans for overdose responses such as naloxone administration and summoning emergency medical services.

Overdose prevention centers are one approach to providing a safer environment for drug use. However, some research suggests that OPCs may be perceived as masculine spaces [29,35]. Women-centered overdose prevention centers and other types of harm reduction spaces can help address the needs of women [36]. There are well-documented barriers to drug treatment among women [18,37]. These include transportation, childcare, fear of child protective services removing children, and drug use stigma. These same barriers may impede women from using overdose prevention centers. In addition to developing new settings for safer drug use, programs to train household members, regardless of whether they use drugs, in opioid overdose prevention and response are also critical. For individuals who engage in solitary drug use, hotlines have been established to assess whether the callers have overdosed and contact local EMS in the event of a suspected overdose [38]. However, the appeal of these programs may be limited to certain populations [39].

### Limitations and Future Directions

Study limitations should be noted. Although we assessed biological sex and sexual orientation, we did not assess gender identity or drivers of drug use settings, such as the intersectional stigma of gender and drug use. Biological sex can differ from gender expression, and trans women face additional structural and symbolic violence. However, in Baltimore populations of people who use opioids, we have found a difference of less than 1% between self-reports of sex and gender. There may also have been differences in the use of settings based on the duration of residence in Baltimore, which was not measured. We also did not assess social network factors that may influence where people use drugs, and recall bias may have influenced the findings. Moreover, we did not assess the use of unsanctioned OPCs. The number of statistical tests may have led to Type 1 errors; however, there was a clear pattern of statistically significant associations. However, the study findings provide strong quantitative evidence of sex differences in the settings where people use drugs, and most participants reported drug use in multiple settings. We also encourage other investigators to qualitatively assess how people choose where to use drugs and why they may avoid or be attracted to certain settings. Harm reduction programs should target drug use settings and provide training and materials to enhance their safety.

## 5. Conclusions

The study findings provide strong quantitative evidence of sex differences in the settings where people use drugs, with women less likely to report drug use in more public settings, and most participants reported drug use in multiple settings. Harm reduction programs should focus on making current drug use settings safer by providing drug use settings with naloxone and HIV/HCV prevention materials and fostering access to additional safer drug use settings, with an emphasis on addressing barriers for women to access setting-focused harm reduction services.

## Figures and Tables

**Table 1:** Sociodemographic and Drug Use Setting Differences by Sex (N = 869).

Variable	Female (346) % (n)	Male (523) % (n)	Total (869) % (n)	χ^2^
Race				7.12 *
African American/Black	64.2 (222)	72.7 (380)	69.3 (602)	
White	28.9 (100)	22.4 (117)	25.0 (217)	
Other	6.9 (24)	5.0 (26)	5.8 (50)	
Education				1.09
≤12th grade/GED	72.0 (249)	75.1 (393)	73.9 (642)	
>12th grade	28.0 (97)	24.9 (130)	26.1 (227)	
Homeless (past 6 months)				2.31
No	59.0 (204)	53.7 (281)	55.8 (485)	
Yes	41.0 (142)	46.3 (242)	44.2 (384)	
Age, years, Mean (SD)	48.6 (11.1)	49.6 (10.9)	49.2 (11.0)	t = 1.78
Setting of Drug Use (past 30 days)				
Your house	83.5 (289)	78.8 (412)	80.7 (701)	3.01
Someone else’s house	68.2 (236)	71.3 (373)	70.1 (609)	0.96
Street	67.9 (235)	78.6 (411)	74.3 (646)	12.42 ***
Alley	54.9 (190)	64.2 (336)	60.5 (526)	7.59 **
Park	44.5 (154)	55.3 (289)	51.0 (443)	9.63 **
Abandoned building	41.9 (145)	50.9 (266)	47.3 (411)	6.70 **
Public restroom	33.8 (117)	39.8 (208)	37.4 (325)	3.16 ^
Car	40.8 (141)	55.4 (290)	49.6 (431)	18.00 ***
Other	8.7 (30)	13.4 (70)	11.5 (100)	4.54 *

Note. Percentages are column percentages; counts shown in parentheses. All percentages represent the proportion within each gender category. ^ *p* < 0.10, * *p* < 0.05, ** *p* < 0.01, *** *p* < 0.001. Age shown as Mean (SD), *t*-value. Percentages may not sum to 100 due to rounding.

**Table 2:** (**a**) Bivariate logistic regression models of drug use settings (N = 869). (**b**) Multivariable logistic regression models of drug use settings (N = 869).

(**a**) Bivariate logistic regression models									
**Predictor**	**Your House** **OR** **(95% CI)**	**Someone Else’s House** **OR** **(95% CI)**	**Street** **OR** **(95% CI)**	**Alley** **OR** **(95% CI)**	**Park** **OR** **(95% CI)**	**Abandoned Building** **OR** **(95% CI)**	**Public Bathroom** **OR** **(95% CI)**	**Car** **OR** **(95% CI)**	**Other** **OR** **(95% CI)**
Female (vs. male)	*1.37 * *(0.96–1.94)*	0.86 (0.64–1.16)	**0.58 ** **(0.42–0.78)**	**0.68 ** **(0.51–0.89)**	**0.65 ** **(0.49–0.85)**	**0.70 ** **(0.53–0.92)**	*0.77 * *(0.58–1.03)*	**0.55 ** **(0.42–0.73)**	**0.61 ** **(0.39–0.96)**
Age	**1.03 ** **(1.01–1.04)**	**0.98 ** **(0.97–1.00)**	**0.94 ** **(0.92–0.96)**	**0.94 ** **(0.93–0.95)**	**0.97 ** **(0.95–0.98)**	**0.95 ** **(0.94–0.96)**	**0.97 ** **(0.96–0.98)**	**0.98 ** **(0.97–0.99)**	*0.98 * *(0.96–1.00)*
Race: White (vs. Black)	0.96 (0.65–1.41)	1.34 (0.94–1.89)	**3.65 ** **(2.31–5.77)**	**5.52 ** **(3.67–8.30)**	**2.36 ** **(1.71–3.26)**	**4.25** **(3.03–5.97)**	**2.43 ** **(1.77–3.34)**	1.03 (0.75–1.40)	1.34 (0.84–2.15)
Race: Other (vs. Black)	0.94 (0.46–1.94)	1.32 (0.69–2.55)	1.61 (0.81–3.21)	**1.85 ** **(1.01–3.40)**	*1.69 * *(0.94–3.02)*	**2.30 ** **(1.28–4.13)**	**2.42 ** **(1.36–4.33)**	*1.72 * *(0.95–3.11)*	*1.91 * *(0.89–4.12)*
Education >12/GED (vs. ≤12/GED)	1.00 (0.68–1.46)	1.00 (0.72–1.39)	1.11 (0.78–1.57)	0.97 (0.71–1.32)	1.19 (0.88–1.61)	1.17 (0.87–1.59)	1.23 (0.90–1.67)	1.06 (0.78–1.43)	**1.70 ** **(1.10–2.64)**
Homelessness (yes vs. no)	**0.37** **(0.26–0.53)**	**2.67 ** **(1.95–3.66)**	**5.61 ** **(3.83–8.23)**	**5.93 ** **(4.32–8.13)**	**4.36 ** **(3.27–5.82)**	**8.61 ** **(6.33–11.72)**	**3.06 ** **(2.30–4.08)**	**1.81 ** **(1.38–2.37)**	**2.89 ** **(1.86–4.50)**
Daily heroin/fentanyl use (yes vs. no)	0.91 (0.60–1.38)	**1.61 ** **(1.15–2.25)**	**1.48 ** **(1.04–2.10)**	**1.89 ** **(1.37–2.61)**	**1.81 ** **(1.30–2.50)**	**1.67 ** **(1.20–2.32)**	**1.88 ** **(1.32–2.68)**	**1.47 ** **(1.06–2.03)**	1.33 (0.78–2.28)
Daily crack/cocaine use (yes vs. no)	1.01 (0.72–1.41)	**2.11 ** **(1.57–2.84)**	**1.88 ** **(1.38–2.57)**	**3.04 ** **(2.29–4.04)**	**2.26 ** **(1.72–2.97)**	**3.11 ** **(2.36–4.10)**	**1.42 ** **(1.08–1.88)**	1.22 (0.93–1.59)	**1.64 ** **(1.07–2.52)**
(**b**) Multivariable logistic regression models									
**Predictor**	**Your Residence** **aOR** **(95% CI)**	**Someone Else’s Residence** **aOR** **(95% CI)**	**Street** **aOR ** **(95% CI)**	**Alley** **aOR ** **(95% CI)**	**Park** **aOR ** **(95% CI)**	**Abandoned Building** **aOR ** **(95% CI)**	**Public Bathroom** **aOR ** **(95% CI)**	**Car** **aOR ** **(95% CI)**	**Other** **aOR ** **(95% CI)**
Female (vs. male)	1.34 (0.93–1.92)	—	**0.49** **(0.35–0.70)**	**0.50 ** **(0.35–0.69)**	**0.57 ** **(0.42–0.78)**	**0.53 ** **(0.38–0.75)**	*0.76 * *(0.56–1.03)*	**0.55 ** **(0.41–0.73)**	**0.59 ** **(0.37–0.94)**
Age	*1.01 * *(1.00–1.03)*	1.00 (0.98–1.01)	**0.96 ** **(0.95–0.98)**	**0.97 ** **(0.95–0.99)**	0.99 (0.97–1.01)	*0.99 * *(0.97–1.00)*	0.99 (0.98–1.01)	**0.98 ** **(0.97–1.00)**	1.00 (0.98–1.02)
Race: White (vs. Black)	—	0.84 (0.55–1.27)	**1.80 ** **(1.06–3.06)**	**2.97 ** **(1.84–4.80)**	*1.41 * *(0.95–2.09)*	**2.43 ** **(1.58–3.74)**	**1.70 ** **(1.17–2.48)**	*0.71 * *(0.49–1.03)*	—
Race: Other (vs. Black)	—	0.93 (0.47–1.85)	0.89 (0.42–1.91)	1.06 (0.53–2.11)	1.11 (0.59–2.10)	1.35 (0.68–2.65)	*1.79 * *(0.97–3.30)*	1.38 (0.74–2.57)	—
Education >12/GED (vs. ≤12/GED)	—	—	—	—	—	—	—	—	**1.71 ** **(1.09–2.68)**
Homelessness (yes vs. no)	**0.42 ** **(0.29–0.60)**	**2.37 ** **(1.69–3.34)**	**3.82 ** **(2.54–5.74)**	**3.78 ** **(2.68–5.34)**	**3.33 ** **(2.44–4.55)**	**6.11 ** **(4.39–8.50)**	**2.44 ** **(1.78–3.33)**	**1.57 ** **(1.16–2.12)**	—
Daily heroin/fentanyl use (yes vs. no)	—	*1.37 * *(0.96–1.95)*	1.25 (0.85–1.85)	**1.51 ** **(1.04–2.20)**	**1.47 ** **(1.03–2.10)**	1.21 (0.82–1.79)	**1.69 ** **(1.16–2.47)**	*1.39 * *(0.99–1.96)*	—
Daily crack/cocaine use (yes vs. no)	—	**1.72 ** **(1.25–2.36)**	1.33 (0.94–1.90)	**2.25 ** **(1.61–3.13)**	**1.72 ** **(1.27–2.34)**	**2.30 ** **(1.65–3.21)**	0.97 (0.71–1.33)	1.14 (0.85–1.52)	1.39 (0.88–2.18)

Notes: OR = odds ratio; aOR = adjusted odds ratio; CI = confidence interval. Cells are bold if *p* < 0.05 and italicized if 0.05 ≤ *p* < 0.10. Reference categories: male; Black (African American/Black); education ≤12/GED; no homelessness in past 6 months; no daily heroin/fentanyl use; no daily crack/cocaine use. Cells with — indicate that the predictor was not included in the multivariable model for that outcome.

## Data Availability

The data presented in this study are available on request from the corresponding author.
